# Drone Brood Homogenate as Natural Remedy for Treating Health Care Problem: A Scientific and Practical Approach

**DOI:** 10.3390/molecules25235699

**Published:** 2020-12-03

**Authors:** Ewelina Sidor, Małgorzata Dżugan

**Affiliations:** Department of Chemistry and Food Toxicology, Institute of Food Technology and Nutrition, University of Rzeszow, Ćwiklińskiej 1a Street, 35-601 Rzeszow, Poland; mdzugan@ur.edu.pl

**Keywords:** drone brood, apilarnil, steroid hormones, therapeutic value, dietary supplement

## Abstract

Drone brood homogenate is a little-known bee product used in folk medicine to treat various health problems. It is a very nutritious milky substance with high content of nutrients: proteins, lipids, fatty acids, carbohydrates, vitamins (A, B, E and D), and minerals. Moreover, when collected on early stage of larvae development, it is, most of all, rich source of sex hormone (testosterone, progesterone and estradiol). Some beekeepers consider drone brood as a waste product, although in some countries they use it to fight *Varroa*. Meanwhile, in many scientific reports a curative effect of bee drone homogenate in treating urgent global health problems have been confirmed, including ovarian dysfunction in women and male infertility, thyroid and immunity disorders, as well as malnutrition in children. A few dietary supplements based on drone brood are available online. Many patents relating to drone brood-based dietary supplements have been filed in Russia, but their prevalence in EU countries is still limited. Further research is needed to fully recognize the pharmacological activity and increase the use of drone brood.

## 1. Introduction

Honey and other bee products, i.e., pollen, bee bread, propolis, royal jelly, and wax have been valued as food stuffs all over the world since ancient times [1,2]. The basis for the comprehensive use of bee products in human nutrition and treatment is their diverse and unique chemical composition, including the content of numerous characteristic substances with a bioactive effect [3]. Bee products are naturally occurring pharmaceutical products with a multidirectional effect on the living organism, including humans. Their pharmacologically active fractions are used in many fields of treatment and pharmacy as pharmacopoeial raw materials, dietary supplements and cosmetics [4,5,6]. The biologically active compounds of bee products include bactericidal agents and antioxidants [7]. Drone brood is one of the bee products that are not commonly known in Europe but is a recognized and frequently used remedy in some countries of the world, such as Romania, China, Zambia, Senegal and Ecuador [8,9,10,11,12].

Drones, as male honeybees, are one of polymorphic forms of bees, next to the queen and worker bees. Drones bite their way out of the drone cell on the 24th day after egg laying [12] and are easily recognisable by their large size, oval body, large eyes and strong wings. They do not have pollen sacs, a wax gland or sting. They appear in bee colony in spring and their only task is to inseminate with a queen during her mating flight. The few drones that do get a chance to mate die after mating.

Drones typically compose 5–10% of the adult population of bee colony during the breeding season from spring to autumn with a peak in the late spring or early summer. A colony might adjust drone production in relation to season (length of day and temperature) and other environmental cues, including size of colony, availability of food as well as colony microclimate [13].

Drone brood homogenate (known as apilarnil or less apistimul) is a bee product obtained by the collection of drone larvae from drone cells, from 3 to 11 days after hatching [14,15]. Apilarnil, which may be defined as the male equivalent of royal jelly, is obtained from drone brood and then freeze-dried. It was discovered by the Romanian apitherapist Nicolae V. Iliesiu. Etymologically, the term originates from “api” for bee, “lar” for larvae, and “nil” as a shortened form of its discoverer’s name, Nicholae Iliesiu [16].

Currently, many studies characterizing the morphological composition, properties and use of drone brood are available [12,16,17,18,19]. The literature lacks a description of the detailed characteristics of the raw material variability which depend on the development stage. In addition, drone brood quickly loses biological activity once it is removed from a wax comb, so fixation strongly determines its effectiveness [20]. Considering the above, the aim of the study was to review the scientific reports regarding the chemical composition variability as well as pharmaceutical potential of the drone brood homogenate. Moreover, the e-market and worldwide patent base was searched in terms of practical application of drone brood as dietary supplements.

## 2. Drone Brood Origin

**Figure 1:**
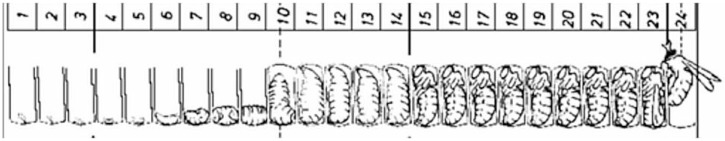
Drone brood development stage [23].

Drone brood is defined as male bees developing in wax comb cells from unfertilized eggs by a process known as parthenogenesis. The development of drones (24 days) is longer than that of queens and workers (16 and 21 days, respectively) [21]. Over the first ten days, the drones develop in an open cell and the larvae are progressively provisioned by workers. The final 14 days of development occur under capping. Workers bees close the cells with queen bees and workers with a cap, while the drone cells are closed with a convex cap. This difference in caps allows for the identification of male brood in the comb. During their time of enclosure within the cell, pre-pupa and pupa do not feed, metamorphosis occurs (Figure 1), and the process of spermatogenesis is completed [22].

In the first days of development (day 1–3), regardless of the polymorphic form, the larva weighs about 0.11 mg [21]. It is only from the 4th day that the body weight and length of the various larvae begin to differ, depending on their polymorphic form. A seven-day-old queen larva weighs 270 mg, a worker larva 80 mg, and a drone larva 120 mg [22,24]. On the day of sealing (day 11) the drone larvae reach a weight of 350 mg; thus, their weight is greater than that of the mother and bee larva [24]. Moreover, they contain more proteins, lipids and sulfhydryl groups. Considering the above and the ease of collecting from comb wells, 11-day-old drone brood is most suitable for technological use [19].

In the field of beekeeping, drone brood is sometimes treated as waste whereas it should be used for the sake of wealth of valuable nutrients and bioactive ingredients. However, the amount of drone brood present in the colony regulates drone production through a negative feedback process [12]; thus, the removal of drones from the colony should upregulate drone production when they are harvested regularly. Current evidence indicates that the practice of drone brood removal is an effective method of controlling *Varroa* spp. [14]. In Europe, particularly in Nordic countries, this technique combined with chemical treatment, is used as part of a *Varroa* spp. trapping strategy [12].

## 3. Stability and Preservation of Drone Brood

Drone brood used for technological processing should be obtained on the appropriate development day. Literature data concerning the best day of collecting material from the comb are strongly divergent [25]. According to Stangaciu and Hartenstein [24] and Sołodenko [26], these should be 6–7 days old larvae. In turn, Czerkasowa and Prochoda [27] have indicated that 7–9 days is optimal, while Budnikowa [14] has indicated that 10–14 days old pupae are best suited for technological processing. This discrepancy is a consequence of taking into account drone brood activity by some authors whereas other focused on the usefulness of material for processing.

Obtaining larvae from the combs may be easier prior to capping as the larvae can then be removed from the combs with a stream of water. For capped brood combs, freezing the comb (at −20 °C or in liquid nitrogen −196 °C) is recommended before proceeding with manual separation. A fast and less labor-intensive method is squeezing the unfrozen or thawed brood combs above a sieve and letting the juices pass through the sieve. However, such juice needs to be frozen or used immediately because it oxidizes extremely quickly [12].

Cutting out the drone larvae from the hive is associated with a loss of biological properties in a very short time. It is important to process the acquired brood within 24 h or to protect it against the loss of beneficial properties. Moreover, drone brood is very sensitive to bacterial activity, so it should be stored in freezer [12,28]. The larvae can be stored for up to 6 days at a temperature of −2 °C, and for up to 10 months at −18 °C without losing their biological properties [29]. It is possible to freeze larvae or homogenate in liquid nitrogen (−196 °C). However, this is a very costly process and is not suitable for small-scale use [29], where usually the honey is used to preserve drone brood. For this purpose, a previously prepared homogenate of drone brood is added to honey in amounts up to 1–2% of the final volume. Storage at room temperature of the final product allows for the maintenance of its properties for 6 months. Kryłow et al. [30] proved that the preservation of the homogenate with honey at a concentration of 3–5%, and storage at 6–12 °C allows for the maintenance of properties for 6 months. An effective method of preserving drone brood is to mix the larvae with 40% ethyl alcohol in a 1:1 ratio [20]. It is also possible to dry the drone larvae. They can be dried with natural sun rays or with the use of air-circulating dryers (70–75 °C). Infrared or heating lamps are also used for drying. This thermal process protects drone brood for 7 months. Deposition on the adsorbent is another method of brood conservation. The combination of glucose and lactose in the ratio of 1:1, and then brood and adsorbent (1:6), allows the product to be stored for 3 years at room temperature, after prior placement of the product in a refrigerator (4–6 °C) for 3 months [31].

## 4. Functional Components of Drone Brood

The chemical composition of fresh drone brood is similar to that of royal jelly. Drone brood homogenate is characterized by a higher water content but its protein and carbohydrate content is lower than that in royal jelly [32,33]. A comparison of the physicochemical and chemical properties of fresh drone homogenate, lyophilizate (apilarnil) and fresh royal jelly is presented in Table 1.

Drone brood homogenate is a milky, dense substance with creamy consistency. Its colour varies from white and yellowish to pale grey [28,36]. It is characterized by a sweet, with a slightly sour taste and distinctive smell similar to that of royal jelly [36,38]. The greatest differences in the physicochemical composition were observed between fresh and freeze-dried brood homogenate in terms of water content.

### 4.1. Proteins and Amino Acids

Fresh drone brood is a rich source of protein compared to other bee products. Lazaryan et al. [35] reports that it constitutes 38.5% of the product, while Bogdanov [41] has determined that it makes up 52.3% of fresh drone brood. In addition, drone brood is a source of 20 amino acids, including 8.7% in the free state, and 15.9% of exogenous amino acids. The essential amino acids, include threonine, valine, methionine, isoleucine, leucine, phenylalanine, lysine, histidine and tryptophan. Among all of the amino acids found in drone brood, the greatest amounts are glutamic acid (6.5% of all amino acids), leucine and aspartic acid (3.6% each), proline (3.4%), lysine (2.9%), valine (2.3%) and alanine (2.1%) which make up about 60% of all amino acids. Taurine and phosphoserine are non-protein amino acids found in drone brood [42]. Similar proportions of the amino acid composition are found in royal jelly. Substance with the highest content (similarly to drone brood) is glutamic acid (8.3%), tyrosine (4.3%), proline (3.9%), aspartic acid (2.8%), leucine (3.0%), lysine (2.9%) and valine (1.6%). A relatively large part of the protein amino acids consists of methionine (3.7%), tryptophan (3.4%) and arginine (3.3%), which are present in drone brood in trace amounts [28].

### 4.2. Lipids

Drone brood is a source of lipids and according to Iliesiu [43] and Stangaciu and Hartenstein [24] they account for 5–8% of the total composition. Slightly different values are given by Barnutiu et al. [28] and Narumi et al. [44]. Similarly, Isidorov et al. [18] showed the lipid content in drone brood at the level of 3.5%. This group of substances includes triglycerides, free fatty acids, fatty acid esters and decenoic acids. The fatty acids include saturated acids (40%), of which palmitic and stearic acids are the most abundant and 50% of the contents were monounsaturated acids where oleic acid constitutes 32.3% of this group. Drone brood is also a source of polyunsaturated fatty acids (10%), of which linoleic and y-linolenic are present in the highest amounts (1.1 and 1.7% respectively). Polyunsaturated acids as essential unsaturated fatty acids must be supplied to the body in a healthy diet [21].

Most of the fatty acids contained in the drone brood exist in the form of esters [30]. This bee product is a relatively large source of glyceryl-1,2-dioleate-3-palmitate ester. Additionally, plant sterols which form a specific group of lipid compounds were found in the brood. Those which occur in the highest amounts are campesterol (5.5 mg/100 g), β-sitosterol (1.3 mg/100 g), stigmasterol (0.2 mg/100 g) and 5-hydroxysitosterol (1.3 mg/100 g) [38]. They belong to a group of phytosterols (plant substances) which have a similar structure to human cholesterol. A regular intake of these components in a diet (about 2 g daily) helps to lower cholesterol effectively, reducing the risk of atherosclerosis and heart attack and protecting against some cancers, as well as prostate hyperplasia [44].

### 4.3. Sugars

**Figure 2:**
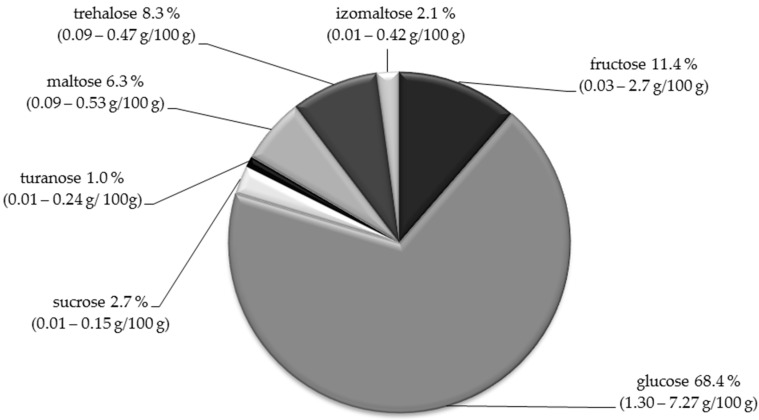
The sugar profile of drone brood homogenate as % share [27]. The range of sugar content in [g/100 g] of the drone brood homogenate is presented in brackets [27].

The comprehensive characterization of sugars present in the brood was carried out by Barnutiu et al. [28]. On the basis of the results obtained, the highest percentage of glucose and fructose was found (68.3% and 11.4% respectively). In the composition of the drone brood other sugars, occurring in smaller amounts, were identified (Figure 2). Similar results were reported by Stangaciu and Hartenstein [24].

### 4.4. Hormones

Drone brood contains two types of hormones: those regulating the development of the larvae and the sex ones. Juvenile and moulting (metamorphosis) hormones influence the development of drone larvae. The juvenile hormone stimulates the growth of drone larvae and inhibits metamorphosis, while the moulting hormone (ecdysone) inhibits the growth of larvae and stimulates shedding and the transformation of larvae into pupae [45]. Moreover, drone brood is a source of both male (testosterone) and female sex hormones (estradiol, progesterone, prolactine) [14,34,46,47].

**Figure 3:**
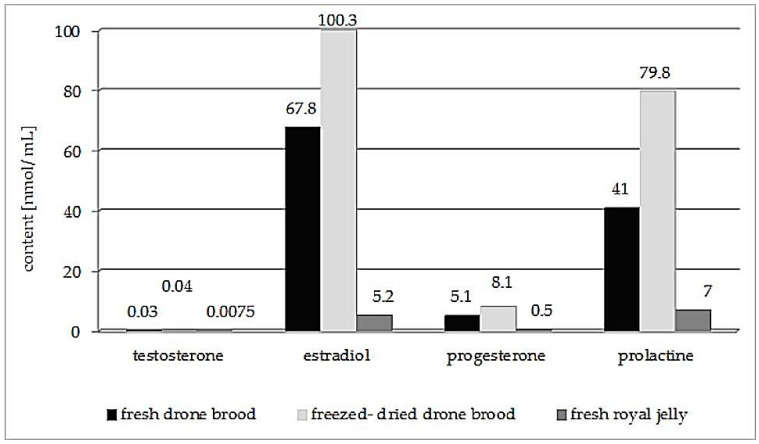
Hormone levels (nmol/mL) in seven-day-old drone brood compared to royal jelly [14,34,47].

Those present in the highest amounts are estradiol and prolactin, while the hormone at the lowest level is testosterone (Figure 3). The content of this male steroid hormone in seven-day-old drone brood is approx. 0.03 nmol/mL. It is four times higher than in the fresh royal jelly. The level of sex hormones in the developing drone larvae varies according to the stage of development [34,41,43], the older larva the higher testosterone and lower progesterone and estradiol levels.

### 4.5. Vitamins and Bioelements

Drone brood is a rich source of both groups of vitamins: water and fat soluble (Table 2). The high content of choline and a-tocopherol is especially noteworthy [27]. In drone brood, relatively large amounts of pantothenic acid and calciferol occur [27,36].

Drone brood is relatively rich in sodium, potassium, calcium, magnesium and phosphorus [47]. The presence of iron, magnesium, zinc, copper, chromium, iodine and selenium was also found. Moreover, according to Sołodenko [26], nickel, gold and silver are present in drone brood.

### 4.6. Antioxidant Activity

Bee products have strong antibacterial and antioxidant properties. The antioxidant components include: polyphenolic compounds, vitamins C and E, enzymes, and other elements [7]. The antioxidant activity of various bee products has been frequently measured using the 1,1-diphenyl-2-picrylhydrazole (DPPH) radical test correlated with total phenolics content (by Folin-Ciocalteu method) (Table 3). Among compared bee products, the highest antioxidant activity was found for propolis, next bee pollen and drone brood homogenate. Moreover, the homogenate of drone brood contains the highest amount of polyphenolic compounds among the tested bee products (Table 3).

## 5. Drone Brood in Scientific Reports

The rich chemical composition of the drone brood contributes to the high degree of biological activity and leads to a beneficial effect on the human body. Due to high protein, vitamins and hormone levels, drone brood effectively prevents the processes of cellular aging and many diseases. This healing effect is widely described by scientists from Romania, Slovakia, Ukraine and Russia [27,31,42]. The in vivo studies carried out to date with the use both animals and humans, indicate the positive effect of drone brood in the treatment of hypothyroidism, liver diseases, it is also used in adaptogenic therapies and in the treatment of infertility (Table 4). In addition, drone brood has antioxidant properties, protects the fetus and increases immunity.

### 5.1. Effects on the Reproductive System and Fertility

The studies aimed at testing the influence of drone brood on the treatment of male infertility was initiated by Iliesiu [43]. As a result of the research, an increase in sexual performance was found, and the functioning of the testicles improved. Consuming the brood has also led to the elimination in some cases of erectile dysfunction. All of the observations were confirmed by tests determining the number of sperm. In another clinical trial [62], infertile men (n = 68) with infertility resulting from the symptoms of sexual neurosis, which is responsible for problems with ejaculation and orgasm. The research carried out by Krylow [30] has shown that drone brood has an androgenic effect and also stimulates the production of testosterone which alleviates sexual disorders.

The androgenic activities of drone brood have been intensively studied in vivo with the use of animals. Seres et al. [63] used an experimental model using rats to test the effectiveness of drone brood on the androgenic functions in males. Increased plasma testosterone levels have been observed in animals receiving drone brood, the same tendency in the weight of the penis glans and seminal vesicle was found. In the control groups (both false control and flutamide application group), no significant changes were observed. Similarly, Bolatovna et al. [47] illustrates that the administration of drone brood solution by injection in pigs increases the weight of the seminal gland and the survivability and mobility of boar sperm. This research also corroborates the idea of the usage of drone brood as a therapy for testosterone deficiency. Experiments with the use of broiler chicks showed the effectiveness of the use of drone brood in the development of secondary sexual characteristics and the length of the comb [64]. It was shown that broilers that received brood with their feed had a longer comb than the control broilers. A similar relationship was observed by Allen et al. [65] who additionally found that broilers which were fed brood showed higher blood testosterone levels.

A few studies reported the usefulness of drone brood in alleviating the symptoms of menopause in woman [43]. It has been shown that drone brood has a supportive effect in neurovegetative disorders in women, with impaired physical and mental fitness, as well as those with a tendency towards depression [30]. Moreover, the use of drone brood by women reduced the initial symptoms of menopause, such as feeling hot, and an increased breathing and heart rate, headaches and dizziness, as well as excessive sweating [66]. The authors emphasize that women using drone brood tolerated it very well.

### 5.2. Adaptogenic Effect of Drone Brood

Drone brood is characterized by an ability to increase the body’s non-specific immunity [53]. Moreover, it improves the physical and mental resistance of experimental animals [30]. It was found that feeding rats with drone brood improved their physical resistance during a swimming test. The experiment also consisted of testing the changes in the level of cortisol in the blood serum of the animals. It was proved that its content in rats subjected to physical activity was 20% higher than at the beginning of the experiment. The results obtained show a clear protective effect against the stress associated with the swimming test. In addition, the obtained results indicate an increase in the mental resistance of the tested animals caused by the drone brood treatment.

Drone brood homogenate was administered in rabbits for two weeks by oral ingestion intraoral intake of 0.6 mL/kg every 48 h (controls treated with gelatin). The experiments showed a decrease of oxidated reaction products in blood and increase of the cell resistance of the drone brood treated rabbits. The thiobarbituric reactive substances (a measure of lipid peroxidation and oxidative stress) decreased by 26%, while that of the controls increased by 25%. At the same time, serum sialic acid concentration (an indicator of glycosylation disorders) decreased in the treated groups by 20% while the controls it increased by 24%. The research conducted proves the influence of drone brood in increasing the immunity of the nervous system to the harmful influence of external factors [58]

## 6. Drone Brood as Dietary Supplement

In many countries (Japan, China, Romania, Russia, Ukraine) brood drones are obtained for preparing snacks in the fried or baked form. They are added to dishes, e.g., instead of toasts for soups or in the form of sauces. Drone brood was used to prepare tinctures, preserves or sweets. Moreover, it is used in the production of dietary supplements and medications [12,67]. However, only a few commercial products were found for sale in Europe online, including Romanian *Apilarnil Potent,* Canadian *ApiDhron*^®^, Slovenian *Femoklim^®^* or Turkish *Harşena Apiterapi Ürünleri*. Meanwhile, many handmade products are offered by apiaries, also as combination with propolis and pollen or honey. All known preparations based on the drone brood, due to extreme chemical instability of its active ingredients, as a rule, contain stabilizing additives, for example, honey, sucrose, lactose or glucose. According to Burmistrova [68], the storability of fresh bee brood can be improved, by binding fresh drone brood to a glucose/lactose adsorbent with the additive of L-ascorbic acid as an antioxidant (50 mg/kg). If the mixture is dried until 4% humidity, product is stable at 4 to 8 °C for 2–3 years [32,68]. Due to the different production technologies (extraction, lyophilization, thermal drying, adsorption, stabilization, mixing), the preparations based on the drone brood have a non-permanent qualitative and quantitative composition, have different severity of nutritional and therapeutic properties, characterized by a different shelf life [16].

The usual daily dose recommend by apilarnil producers for adults is about 300 mg, which can be enlarged to 600–900 mg, if necessary [38]. However, toxicological studies are completely lacking in the available literature, nor adverse effect or toxic dose has been established. The only one study conducted in Russia reports the incidence of allergy towards drone brood application as 2.4% of tested population (*n* = 41) [69].

Conducting the research allowed to patent the recipes for supplements based on drone brood recommended to support the functioning of the organism. To find such practical solutions we used the worldwide Espacenet base which offer free access to over 120 million patent documents. The result list of advanced search for key words combination “drone” and ” brood” given 247 results, when the combination “drone” and ” brood” and “supplement” was used the results were limited to 75. The most interesting current applications which were filed within last 10 years, were summarized in chronological order in Table 5.

The patented supplements are mainly recommended for the prevention and treatment of hormonal disorders in women and men or in a complex with calcium—for osteoporosis and arthritis. The proposed administration amount is very divergent, from 10 to 1000 mg per day. The majority of inventions come from Russia and are applied mainly by one company “Parapharm”, the manufacturer of innovative food supplements and ingredients, which nowadays possess 110 patents of Russia and foreign countries [70].

## 7. Conclusions

Drone brood is a rich in nutrients, little-known bee product which exhibits many beneficial healing and therapeutic properties. Since ancient times, it has been used as a cheap, safe and effective natural remedy against different diseases. Some of the biological and therapeutic effects of drone brood have been confirmed by performing laboratory and animal or human in vivo experiments. Unfortunately, scientists have only just begun to discover the many health benefits of consuming this little-known bee product. Only a few dietary supplements can be found in on-line sale. The dietary supplements based on drone brood were the subject of some filed patent coming mainly from Russia. Meanwhile, due to its high degree of hormonal activity, drone brood should be thoroughly examined in order to be safe used as a component of widely accepted pharmaceuticals in the future. Due to reported allergy incidents toxicological aspects of excessive use of drone brood should be considered.

## Figures and Tables

**Table 1:** Physical properties and chemical composition of raw drone brood homogenate in comparison with the apilarnil product [17,34,35,36,37,38] as well as royal jelly [35,38,39,40].

Characteristic	Quality Parameters		
	Raw Homogenate, Preserved Homogenate	Lyophilizate (Apilarnil)	Fresh Royal Jelly
Sensory properties	dense, cream-like liquid of yellow colour with characteristic odour	amorphous powder of beige to yellow colour with characteristic odour	creamy texture, odourless, but with a distinctive, bitter and tart taste
Solubility	sparingly soluble in water and ethanol practically insoluable in ether		dissolves well in ethyl alcohol, acetone and ether
**Chemical Composition [%]**			
Proteins	7.2–10	9–12	14.6–18.3
Carbohydrates	6.9–8.0	6–17	8.5–10.8
Lipids	3.1–5	5–8	3–8
Ash	3.00	3.00	0.8–3.0
Water content	78.5	3.5	61.0–65.2
Dry matter	25–29	25–35	32.6
pH	5.5–7.5	4.5–6.5	3.8–4.0
Acidity [M/l]	0.7–1.10	-	3.7–4.4
Conductivity [µS/cm]	144.0–178.0	-	194.0–219.0

**Table 2:** Vitamins and bioelements present in powder drone brood [4,15,34,38,44].

Vitamin	Content in Drone Brood [mg/100 g]	Bio Elements	Content in Drone Brood [mg/100 g]
Thiamine (vit. B1)	2.3–4.1	Sodium (Na)	41–900
Riboflavin (vit. B2)	0.1–3.8	Potassium (K)	140–656
Nicotinic acid (vit. B3)	0.3–15.8	Calcium (Ca)	47–126
Choline (vit. B4)	44.3–68.1	Magnesium (Mg)	20–424
Pantothenic acid (vit. B5)	2.6–13.4	Phosphorus (P)	189–330
Pyridoxine (vit. B6)	0.2–0.5	Iron (Fe)	2.4–3.2
Retinol (vit. A)	0.01–0.05	Manganese (Mn)	0.1–4.4
β-carotene (provit. A)	0.03–0.9	Zinc (Zn)	1.3–6.5
α-tocopherol (vit. E)	0.4–1.6	Copper (Cu)	0.6–2.4
Calciferol (vit. D)	0.4–0.6	Selenium (Se)	0.01–0.02

**Table 3:** Total phenolic compounds and antioxidant activity (DPPH) of the bee products [19,48,49,50].

Bee Products	TPC [mg GAE/ kg]	DPPH [%]
Rape honey	254.2	21.8
Multifloral honey	490	39.9
Coniferous honeydew	585.9	66.8
Propolis	1610	96.1
Bee pollen	2340	89
Royal jelly	171	76
Drone brood homogenate	8340	81.6

**Table 4:** Scientific research focused on the use of drone brood in the treatment of various health problems.

Therapeutic Action	Effects	Biological Model	References
Fertility problems and libido reinforcing agent	Alleviates the effects of disorders associated with the menopause in women, increases the body’s immune resistance, increases mental immunity, alleviates symptoms such as: increased heart rate and breathing, feeling hot, excessive sweating	Humans	[30,43]
	Improvement in reproductive properties, increase in the weight in the seminal gland by nearly 22%, increase in the rate of recovery of sexual function, improvement in sperm quality, i.e., ejaculation volume, survival and mobility	Boars, rams	[47,51]
Decrease in cholesterol and triglycerides levels	Lowering the level of cholesterol and triglycerides after two months of use	Rats	[52]
Hepatoprotective activities and stimulates the immune system	Lowering the level of alanine transaminase, alkaline phosphatase and bilirubin in the blood serum	Rats	[53]
	DNA obtained from drone brood protects liver tissue against the hepatotoxic effects of acetylsalicylic acid, buserelin and carbon tetrachloride	Rats	[54]
Fetal shielding properties	Protect the fetus against the harmful effects of acetylsalicylic acid	Rats	[55]
Healing effect in nervous and mental diseases	Improves the mental state of patients with depressive neurosis, fatigue, anorexia, feelings of helplessness	Humans	[43]
	Positive effect on the symptoms of neurastemia (fatigue, weariness, dizziness)	Humans	[56]
	Improvement in memory, reduction of psychomotor lability, return of sphincter control	Humans	[43]
	Improvement of neurovegetative and sexual functions in elderly people	Humans	[32]
Antiatherosclerotic effect	The administration of lyophilized brood administered to rats for two months decreased the content of cholesterol by nearly 50%, triglycerides by 25% and ß-lipoproteins by over 30% in the blood serum compared to the control group.	Rats	[52]
Thyroid disorders	The concentration of thyroxine and triiodothyronine increased by 40%, while the thyroid stimulating hormone decreased by 37% after 30 days of brood administration	Dogs	[57]
Antioxidant effect	Lowers the content of unoxidized substances in blood serum of animals fed drone brood by about 25% compared to control animals	Rats	[58]
Malnutrition in children	Consumption of several doses resulted in weight gain, improvement in general health and appetite, as well as tissue tension.	Humans	[43]
Anaemia	Administration of freeze-dried drone brood to mutant mice with hereditary haemolytic anemia increased their survival rate. The mouse survival was increased from 2 weeks to 7 months in 50% of the animals	Mutant mice	[59]
Other disease	Means of treating urinary tract diseases, diabetes, diarrhea, relieving chest pain, migraines, constipation and to prevent stretch marks on the skin of pregnant women.	Humans	[24,60,61]

**Table 5:** Patents relating to the use of drone brood as an ingredient in supplements in the prevention/treatment of various health problems [https://worldwide.espacenet.com/].

No	Title	Applicants [Country]	Publication Number	Earliest Priority/Publication	Description
1	Insect protein-containing preparation and method for treating androgen deficiency in women	PARAFARM [RU]	UA140992U	2016-03-10/2020-03-25	The preparation for androgen deficiency in women contains a homogenate of brood and can be produced in the form of tablets, capsules or powder. The method of treating androgen deficiency in a woman is to administer the preparation in an amount of 10 mg to 600 mg per day.
2	Method of treating androgen deficiency in women	Kurkus’ N.V. [RU] and 15 co-authors	US20170065646A1	2017-03-09/2018-05-22	The invention relates to biologically active food supplements and is intended for treating androgen deficiency in women and for preventive action against the states associated with osteoporosis. Powdered calcium carbonate mix with drone brood lyophilizate (9:1) in form of tablets. One capsule administration 3 times a day for the month separately from meals.
3	Method of producing bio preparation with nootropic activity	Budget Institution of Higher Vocational Education of the State Fed [RU]	RU2609872C1	2015-11-25/2017-02-06	The bio preparation of homogenizing bee brood drone larvae in cooled isotonic NaCl solution, boiling male bee brood larvae homogenate in order to denature proteins, removing sediment on a paper filter and cleaning with a membrane. The described method allows to obtain a peptide preparation with nootropic effect.
4	Method of treating osteoarthrosis	PARAFARM [RU]	RU2593018C1	2015-10-28/2016-07-27	The invention “Osteomed forte” is using for the invention enables the reduction of the pain syndrome and motor dysfunctions in the joints of the hands and feet. Containing 20–1000 mg of drone brood in combination with vit. D in the amount of 100-50,000 IU, a calcium compound 40–1000 mg. Osteomed forte is taken in the amount of 1 tablet in the morning and evening, with 3-month treatments 3 times a year.
5	Composition for preventing and healing compromised bone and a method of making same	Andreeva E. S. [RU] and 17 co-authors	US2016339063A1	2009-11-30/2016-11-24	The biologically active additive composition comprises the selected pharmacologically active calcium compound in an amount of 16.67–93.75 wt.% and drone brood in an amount of 6.25–83.33% by weight. The application prevents and treats osteoporosis, bone fractures and cavities, arthritis, arthrosis, periodontitis, with a significantly reduced risk of calcium deposition in soft tissues.
6	Application of adsorbed drone brood homogenate and group D vitamins and/or their active metabolites for prevention and treatment of acute respiratory diseases and flu	PARAFARM [RU]	RU2013130302A RU2564111C2 RU2564111C9	2013-07-03/2015-01-08	The proposed biologically active additive comprises adsorbed drone brood homogenate, the daily dose thereof lying between 75 mg and 500 mg, and vitamin or vitamins of group D and/or their active metabolites, a daily dose thereof between 25 IE and 50,000 IE.
7	Biologically active food additive for the prophylaxis of erectile dysfunction in men	PARAFARM [RU]	EP2687107A2	2012-10-04/2014-01-22	The product contains L-arginine and also drone brood with the following components: 50–96.2% by weight of L-arginine and 3.8–50% by weight of drone brood.
8	Coffee drink	PARAFARM [RU]	CA2840708A1 CA2840708C	2011-07-05/2013-01-10	Intended for prophylaxis and therapeutic prophylaxis in conditions associated with fatigue, and is in the form of a coffee or other drink and consists of: 1 part drone brood, 3–9 parts glucose and/or fructose, and 1–100 parts fillers (coffee or other drink in powder form).
9	Preparation and method for the prophylaxis and treatment of atypical osteoporosis	PARAFARM [RU]	US2015224150A1 US9827273B2	2012-04-19/2013-10-24	The preparation consists of 10 mg to 1000 mg daily of drone brood and 50 IU to 100,000 IU daily of vitamin D or vitamins of this group and/or their active metabolites can be supplied in a powder, tablet or capsule.
10	Medication for treatment and prevention urogenital system diseases in males	PARAFARM [RU]	RU2423142C1	2009-12-02/2011-06-09	Preparation contains drone brood with the following weight ratio of components: drone brood (powder) 25–75% naked licorice root (powder) 75–25%. Taking the drug stimulates the work of the glands, normalizes the reproductive system in men, has anti-inflammatory and anesthetic properties, normalizes urination and restores sexual functions.
